# The Role of the *st313-td* Gene in Virulence of *Salmonella* Typhimurium ST313

**DOI:** 10.1371/journal.pone.0084566

**Published:** 2014-01-03

**Authors:** Ana Herrero-Fresno, Inke Wallrodt, Pimlapas Leekitcharoenphon, John Elmerdahl Olsen, Frank M. Aarestrup, Rene S. Hendriksen

**Affiliations:** 1 Department of Veterinary Disease Biology, Faculty of Health and Medical Sciences, University of Copenhagen, Frederiksberg, Denmark; 2 WHO Collaborating Centre for Antimicrobial Resistance in Food-borne Pathogens and EU Reference Laboratory for Antimicrobial Resistance, National Food Institute, Technical University of Denmark, Kgs. Lyngby, Denmark; Queen's University Belfast, United Kingdom

## Abstract

Multidrug-resistant *Salmonella enterica* serovar Typhimurium ST313 has emerged in sub-Saharan Africa causing severe infections in humans. Therefore, it has been speculated that this specific sequence type, ST313, carries factors associated with increased pathogenicity. We assessed the role in virulence of a gene with a yet unknown function, *st313-td,* detected in ST313 through comparative genomics. Additionally, the structure of the genomic island ST313-GI, harbouring the gene was determined. The gene *st313-td* was cloned into wild type *S.* Typhimurium 4/74 (4/74-C) as well as knocked out in *S.* Typhimurium ST313 02–03/002 (Δ*st313-td*) followed by complementation (02-03/002-C). Δ*st313-td* was less virulent in mice following i.p. challenge than the wild type and this phenotype could be partly complemented *in trans,* indicating that *st313-td* plays a role during systemic infection. The gene *st313-td* was shown not to affect invasion of cultured epithelial cells, while the absence of the gene significantly affects uptake and intracellular survival within macrophages. The gene *st313-td* was proven to be strongly associated to invasiveness, harboured by 92.5% of *S.* Typhimurium blood isolates (n = 82) and 100% of *S.* Dublin strains (n = 50) analysed. On the contrary, *S.* Typhimurium isolates of animal and food origin (n = 82) did not carry *st313-td*. Six human, non-blood isolates of *S.* Typhimurium from Belarus, China and Nepal harboured the gene and belonged to sequence types ST398 and ST19. Our data showed a global presence of the *st313-td* gene and in other sequence types than ST313. The gene *st313-td* was shown to be expressed during logarithmic phase of growth in 14 selected *Salmonella* strains carrying the gene. This study reveals that *st313-td* plays a role in *S*. Typhimurium ST313 pathogenesis and adds another chapter to understanding of the virulence of *S.* Typhimurium and in particular of the emerging sequence type ST313.

## Introduction

Worldwide, nontyphoidal salmonellae (NTS) are a major cause of foodborne illness and commonly cause self-limiting gastroenteritis. The ubiquitous *Salmonella enterica* serovar Typhimurium, which affects both humans and animals, is one of the most commonly reported NTS serovars [1]. Thus, globally, it ranked as the first (North America and Oceania) and the second (rest of the world) most common serovar in the period from 2007 to 2011 [1].

Bloodstream or focal infections caused by NTS are infrequent in developed countries [2] and mainly occur in individuals with specific risk factors [3]. In developing countries, NTS frequently cause severe illness, invasive infections, and even death especially among young children with underlying diseases or among HIV-infected immunocompromised adults [4], [5]. NTS are among the most common bacterial pathogens involved in invasive disease in sub-Saharan Africa, causing bacteraemia and meningitis with a mortality rate of 20–25% [3], [6]–[9]. Recently, a highly invasive multidrug-resistant *S.* Typhimurium of a distinct MultiLocus Sequence Type (MLST), ST313, has emerged in several African countries and represents a major public health concern [10]–[12].

Next Generation Sequencing (NGS) studies have revealed that *S*. Typhimurium ST313 is a clonal clade presently circulating in sub-Saharan Africa introduced by a common ancestry more than 50 years ago [13]. The clade has recently been divided up into two lineages which have acquired similar resistance genes on separate occasions [14]. Genomic degradation was identified in the sequenced genome of *S.* Typhimurium ST313 D23580 compared to other *S.* Typhimurium chromosomes [10]. This included the presence of pseudogenes, deletions (some linked to virulence) and a novel repertoire of prophage elements. Kingsley *et al.* [10] suggest that the evolution of the auxiliary genome of *S.* Typhimurium may be of clinical significance, increasing the invasiveness of the bacteria. Additionally, such partial selective genome degradation could potentially also lead to host adaptation to humans as described for host restricted serovars such as *S*. Typhi, *S*. Paratyphi A and *S*. Gallinarum [10]. For these reasons, *S.* Typhimurium ST313 D23580 has been extensively used in studies on host pathogen interaction to gain more knowledge about NTS infection and to elucidate the reason for the apparent increased pathogenicity of this clone [15]–[19]. Some of these studies suggest that *S.* Typhimurium ST313 might have adapted to occupy an ecological and immunological niche provided by HIV, malaria and malnutrition in Africa [11].

Several works have linked *S.* Typhimurium ST313 to invasive disease [10]–[21], however, none has so far identified potential virulence determinants responsible for this disease. Virulence factors of *S.* Typhimurium are conveniently studied in the mouse model, where *S.* Typhimurium causes a systemic typhoid-like infection. Additionally, various *in vitro* cell culture models have proven useful for the analysis of molecular mechanisms during host-pathogen interaction in *S. enterica* infections [22].

The pathogenesis triggered by *S*. Typhimurium has been extensively studied over the last few years and genome studies, involving the application of cutting-edge NGS technologies, have been highly useful for a better understanding of differences between strains. So far, genes encoded from *Salmonella* pathogenicity islands (SPIs) have been reported as the most significantly factors contributing to host cell interactions [23], [24]. Additional virulence determinants, such as those encoded within the pSLT virulence plasmid, adhesins, flagella, and biofilm-related proteins, are also under study and have been found to be involved in several stages of the disease [25]–[29].

In a recent work we have identified a putative virulence associated gene designated *st313-td* (924 pb) through comparative genomics. This gene, of unknown function and unique to *S.* Typhimurium ST313 and *S.* Dublin, is harboured in a putative uncharacterized genomic island termed ST313-GI of ∼18 kb in the *S.* Typhimurium ST313 genomes. Among the genes of unknown function contained in ST1313-GI, *st313-td,* was the only one present in both, ST313 and Dublin chromosomes [12].

The aim of this study was to assess the role of the gene *st313-td* from *S.* Typhimurium ST313 in virulence. In addition, the presence, frequency as well as the expression of the putative virulence gene was estimated in strains of *S.* Typhimurium and *S.* Dublin from a global strain collection. Furthermore, the genomic structure of the genomic island ST313-GI in *S.* Typhimurium ST313 was revealed.

## Materials and Methods

### Ethical statement

The clinical investigation has been conducted according to the principles expressed in the Declaration of Helsinki. Mice infection studies were performed with permission to John Elmerdahl Olsen from the Danish Animal Experiments Inspectorate, license number 2009/561–1675.

### Bioinformatics analyses

Open reading frames (ORFs) of the genomic island ST313-GI were determined using the gene finder named Prodigal [30]. Information available at NCBI **(** www.ncbi.nlm.nih.gov) and the Uniprot database (www.uniprot.org) were used to determine the putative role of the ST313-GI encoded proteins. The analysis of the GC content and map construction of ST313-GI were performed with the Clone Manager Suite 9.0 software.

### Bacterial strains and growth conditions

From a global strain collection maintained at the National Food Institute of the Technical University of Denmark (DTU-Food), a total of 295 *S.* Typhimurium and 50 *S.* Dublin isolates from 10 countries were analysed for the presence of the gene *st313-td* (Table 1). Eighty-two (27.8%) out of the 295 *S.* Typhimurium strains were associated to bacteraemia (isolated from blood specimens) whereas the remaining isolates (72.2%) originated from other sources such as food, stools etc.

**Table 1:** *Salmonella* strains analysed for the presence of *st313-td*.

*Salmonella* serovar (N)	Origin /specimen^a^ (N)	Country of origin^b^ (N)	*st313-td* (N)
*S.* Typhimurium (295)	Human blood (82)	Congo (74), Mauritius (6), Zambia (2)	76
	Human stools (117)	Belarus (35), Mauritius (26), China (56)	39
	Animal (73)	Thailand (30), Denmark (43)	0
	Food (9)	Mauritius (5), Thailand (1), Ethiopia (3)	0
	Others (14)	Nepal (9), Thailand (4), Congo (1)	6
*S.* Dublin (50)	Human (25)	Denmark (50)	50
	Cattle (25)		

All the strains were collected between the years 2000 and 2011. Isolates were serotyped in Denmark or Thailand as previously described [35], [55], [56]. The strain collection is conserved at DTU –Food (Technical University of Denmark).

^a^ The animals strains were collected from poultry (N = 50), pig (N = 22) and shrimp (N = 1).

^b^ 29 out of the 82 isolates from Congo were previously described [56]. 16 strains from Mauritius (N = 1 from blood, N = 2 from food and N = 13 from stools) were associated to the same outbreak [57]. The isolates from Belarus, China and Nepal (all them isolated from water) were also published elsewhere [33], [34], [35].

*S.* Typhimurium 02–03/002 was used in all the experiments conducted in this study representing the clone *S.* Typhimurium ST313. This strain was isolated from a bacteremic child in the region of Lwiro (Democtratic Republic of Congo) in 2002–2003 [12]. The *S.* Typhimurium non-ST313 strains, 4/74 and 14028, were used as controls in the different assays. The virulence properties of 4/74 and 14028 have been previously described [31], [32].

The isolates were inoculated in Luria-Bertani broth or plates (LB) (Oxoid) and incubated for 16 h at 37°C. In addition to these conditions, broths were shacked at 200 rpm during incubation. If necessary, the LB medium was supplemented with antimicrobials at the following concentrations: kanamycin (50 µg/mL), apramycin (75 µg/mL), gentamicin (25 µg/mL) (Sigma).

### Detection of the gene *st-313-td* in S. Typhimurium and S. Dublin strains

The presence of the gene *st313-td* was evaluated by PCR using the primers st313-td-F and st313-td-R (TAG Copenhagen A/S) (Table S1). The following conditions were used for the amplification: 5 min at 94°C; 30 cycles of 1 min at 94°C, 30 sec at 55°C, and 1 min at 72°C; terminating with one cycle of 10 min at 72°C. The isolate 02–03/002 served as positive control, providing an amplicon of 1029 pb, whereas 4/74 was used as negative control.

### Whole genome sequencing, multilocus sequence typing and identification of Single Nucleotide Polymorphisms (SNPs)

Six *S*. Typhimurium strains collected in Belarus (*S.* Typhimurium 1093 and *S.* Typhimurium 133, isolated from stools) [33], China (*S*. Typhimurium 2006–130 and *S.* Typhimurium 2007–024, from stools) [34], and Nepal (*S*. Typhimurium B1 and *S.* Typhimurium D12, from water) [35] (Table 1) harbouring the gene *st313-td,* were sequenced by Illumina GAIIx genome analyzer (Illumina, Inc., San Diego, CA). The raw reads were subsequently submitted to the European Nucleotide Archive (www.ebi.ac.uk/ena/) under accession no. ERP004195. The raw reads can be accessed freely from the following link; http://www.ebi.ac.uk/ena/data/view/PRJEB4869. Assembly was performed by using the pipeline available on the Center for Genomic Epidemiology (www.genomicepidemiology.org). The latter is based on Velvet; algorithms for *de novo* short reads assembly [36].

MultiLocus Sequence Typing (MLST) was performed on the six isolates according to MLST Databases: http://mlst.ucc.ie/.

SNPs were identified as previously described [12] using the *S.* Typhimurium ST313 D23580 genome as a reference. A SNP tree showing the divergence/relatedness of the *Salmonella* Typhimurium strains carrying *st313-td*, assigned to different ST types is shown in Figure S1.

### Expression studies

Bacteria were grown to logarithmic phase in LB (OD_600nm_  = 0.4±0.01). RNA was isolated from 1.5 ml aliquots of overnight cultures by mechanical disruption with the FastPrep system (Bio101; Q-biogene) and by using the RNeasy mini kit (Qiagen). Quantity and quality of total RNA was determined with the NanoDrop 1000 spectrophotometer (Thermo Scientific). The RNA was DNase treated with the TURBO™ DNase and reverse transcribed with the High Capacity cDNA Reverse Transcription Kit (Applied Biosystems) following the supplier's recommendations. The qPCR was done with the Maxima SYBR Green/Rox qPCR Master Mix and gene specific oligonucleotides (Table ) in a MxPro3000 cycler. The gene *rsmC* [37] was used as reference.

### Construction of strains and plasmids

The wild type strains (WT) and plasmids used for the cloning and mutagenesis experiments as well as the mutated isolates constructed are listed in Table 2.

**Table 2:** Strains and plasmids used for mutagenesis and derived isolates.

Strains and plasmids	Relevant features	Reference
***S.*** ** Typhimurium strains**		
02–03/002	Wild type ST313, *st313-td*-positive strain	12
4/74	Wild type. Virulent reference strain	31
14028	Wild type. Virulent reference strain	ATCC
KP1274	Restriction deficient strain	41
02-03/002-E	02–03/002, pACY177, Kn^R^	This work
Δ*st313-td*	Δ*st313-td*, Apr^R^	This work
02-03/002-C	Δ*st313-td*, pACY177+*st313-td* (pAHF4), Apr^R^, Kn^R^	This work
4/74-E	4/74, pACY177, Kn^R^	This work
4/74-C	4/74, pACY177+*st313-td* (pAHF4), Kn^R^	This work
*invH*201::Tn*phoA*	*invH* mutant, Kn^R^	46
Δ*ssaV*	*ssaV* mutant, SPI2-T3SS defect, Kn^R^	45
**Plasmids**		
pACY177	Cloning vector, Ap^R^_,_ Kn^R^	40
pAHF4	pACY177 expressing *st313-td*	This work
pKD46-Gn	Plasmid with λ red recombinase expressed from arabinose inducible promoter and gentamicin resistance gene *aac(3)-Id*	39
pUO9090	Apr^R^	Unpublished

The mutagenesis of 02–03/002 for the gene *st313-td* was performed using Lambda Red Mediated Recombination as previously described by Datsenko and Wanner [38] leading to the isogenic strain Δ*st313-td*. Since the WT strain was resistant to ampicillin, a derivative conferring gentamicin resistance (encoded by the gene *aac*(3)-*Id*) of the helper plasmid pKD46 was used [39]. Selection of mutants was performed on LB agar plates supplemented with apramycin (75 µg/mL). Apramycin selection was carried out because the isolate 02–03/002 harbors the gene *aac(6*′*)-Iaa* (conferring resistance to Kanamycin). The apramycin resistance-encoding gen (*aac(3)IV*) was amplified from the plasmid pUO9090 (M.C. Martín, unpublished results). The resulting construct, Δ*st313-td*, was verified by PCR for the absence of the gene *st313-td.*

The PCR amplicon corresponding to the gene *st313-td* obtained from 02-03/002 was cloned into the restriction endonuclease site *Sca*I-*Pst*I of the vector pACY177 [40] giving rise to the plasmid pAHF4. The promoter of the Ampicillin cassette present in the vector was used to ensure the expression of *st313-td.* The presence of the insert was confirmed by PCR and restriction endonuclease assays. The restriction deficient strain *Escherichia coli* KP1274 was used as primary recipient for plasmids [41]. Competent cells of KP1274 were prepared and electroporated according to standard conditions [42] using the recombinant vector mentioned above and the pACY177 insert-free plasmid. Transformants were selected from LB agar plates containing kanamycin (50 µg/mL) and plasmids with and without the insert were purified and re-electroporated into the competent strain of 4/74. The selection of the transformants was conducted as previously indicated and the resulting mutated strains were named 4/74-E (harbouring the insert-free plasmid pACY177) and 4/74-C (containing the recombinant vector pAHF4). Both constructs were verified by plasmid analysis, restriction endonuclease assays and PCR. The primers used for the cloning and mutagenesis of *st313-td* are listed in Table S1.

After being recovered from KP1274 competent cells, the plasmid pAHF4 was also electroporated into Δ*st313-td* competent cells in order to obtain the complemented strain 02-03/002-C.

### Analysis of the growth of WT and genetic manipulated strains in LB

The WT and the mutated strains (Table 2) were assessed for the ability to grow in LB media. The isolates were grown at 37°C, 200 rpm for 16 h in LB before sub-culturing into fresh media at a 40 fold dilution and further grown with assessments every 15 min for 18 h using Bioscreen C. A growth curve for each of the strains was obtained and growth was similar between the mutant and WT strains reaching the stationary phase after approximately 6–8 h post-inoculation (not shown).

### Mouse mixed infections

Infections of female six-week old C57/BL6 mice were performed as described by Jelsback *et al*. [43]. Briefly, groups of five mice each were inoculated i.p. with 0.1 ml of a 1∶1 mixture of the WT and the mutated bacteria grown to stationary phase, suspended in physiological saline. The inocula were prepared as follows; WT and mutated isolates were grown for 16 h, 200 rpm at 37°C in LB. The WT and mutant strains were mixed before the infection to provide a challenge dose of 5×10^3^ bacteria of each strain. The CFU and ratio between WT and mutated isolates were enumerated by plating as described [43]. Mice were killed at six days post-inoculation by cervical dislocation. Severely affected animals were sacrificed earlier for animal welfare reasons, but otherwise treated as the rest of the group. The spleens were removed aseptically and bacteria recovered. Ten-fold dilutions series were prepared and plated on LB agar plates and CFU counts were performed. One hundred colonies were randomly picked and tested for antimicrobial resistance to the relevant compounds (Apramycin 75 µg/mL or Kanamycin 50 µg/mL) to determine the proportion of the mutant strains. The competitive index (CI) was calculated based on the ratio of the mutant/WT from the spleen in relation to the mutant/WT ratio of the inoculum. A CI = 1 indicates that the virulence of the strains tested is equal. A CI<1 shows that the mutant is less virulent than the WT.

### Infection of macrophages

The role of the gene *st313-td* in the intracellular survival and replication within mouse macrophages was investigated using J774.1 cells; a mouse monocyte-derived macrophage-like cell line as previously described [44] with modifications. The isolate 14028 and the isogenic strain Δ*ssaV* were used as positive and negative control for the infection assays, respectively. The latter is deficient in *ssaV,* a structural component of the SPI2-encoded T3SS [45] reducing its rate of intracellular replication with regard to the WT.

Briefly, macrophage cells were cultured in RPMI+GlutaMAX^TM^-I, Earles, 25 mM HEPES (Gibco) supplemented with 10% (v/v) heat-inactivated FBS and 25 µg/mL gentamicin. Cells were incubated in a humidified 37°C, 5% CO_2_ incubator. The bacteria were grown in LB to a stationary phase, harvested at 8000 rpm for 5 min and resuspendeded in 0.9% (w/v) NaCl. Complement-opsonisation of the bacteria prior to infection, if used, was performed by mixing the bacteria with mouse serum followed by incubation at 37°C for 20 min. Bacteria were added at a multiplicity of infection of 10∶1 (bacteria complement-opsonized) or 50∶1 (bacteria not opsonized). Monolayers were centrifuged at 300 rpm for 5 min immediately after addition of the bacteria followed by incubation for 25 min at 37°C, 5% CO_2_. Enumerations of the bacteria in the inoculum were verified by plating onto LB agar plates (counted before infection). After infection, the media was removed and monolayers were washed twice with 0.9% NaCl. At this point (defined as time 0 h) fresh media containing 250 µg/mL amikacin was added to kill the extracellular bacteria. The plates were incubated for 1 h at 37°C, 5% CO_2_ before new media supplemented with 100 µg/mL amikacin was added for the remaining part of the experiment. This high concentration was used to ensure the killing of extracellular bacteria (lower concentrations did not always kill 100% of the extracellular population after 20 h). To enumerate the bacteria, cells were washed twice with 0.9% NaCl, subsequently lysed in 1 ml 0.1% Triton X-100 (v/v). The viable intracellular bacteria were enumerated by colony counts of lysate dilutions plated on LB agar plates. For intracellular survival, bacteria were enumerated at t = 1 h (survival 1 h post-uptake), and t = 20 h (survival/replication 20 h post-infection). When opsonization was not performed, only survival after 1 h post-uptake (t = 1 h) was measured. Values determined at t = 20 h were expressed relatively to CFU for the WT determined at T1. The experiments were carried out in quadruplicates.

### Macrophage cytotoxicity assay

Cytotoxcity was assessed by measuring the release of cytosolic lactate dehydrogenase (LDH) into the supernatants as previously described [44] with modifications. J774 cells were incubated with bacteria for a 25 min infection period and then cultured in medium containing 250 µg/mL amikacin for 1 h and 100 µg/mL for the remaining part of the assay (as described for the infection assays). At both times, t = 1 h and t = 20 h post-uptake, the culture supernatants of infected cells were collected, transferred to 96-well plates, and their LDH levels were measured using the colorimetric Cytotox 96 kit (Promega). The relative LDH release was calculated as 100× (experimental release – spontaneous release)/(total release – spontaneous release) where spontaneous release is the amount of LDH activity in the supernatant of uninfected J774 cells and total release is the activity in cell lysates.

### Infection of epithelial cell lines

The association between the gene *st313-td* and the invasion of the human epithelium was analysed using Int-407 cells (HeLa-derived epithelial cells). The isolate 4/74 and the isogenic strain *invH*201::Tn*phoA* [46] were used as positive and negative control for the infection assays, respectively. The latter has a mutation in the *invH* gene, reducing its rate of invasion with regard to the WT strain.

The epithelial cells were cultivated in “Eagle Minimum Essential medium” MEM+ GlutaMAX^TM^-I, Earles, 25 mM HEPES (Gibco) supplemented with 10% (v/v) heat-inactivated fetal bovine serum (FBS, Invitrogen) and 25 µg/mL gentamicin to prevent bacterial contamination. The cells were grown statically in a humidified 37°C, 5% CO_2_ incubator. Twenty-four hours prior to infection, the Int-407 cells were seeded in 24-well plates in a concentration of 5×10^5^ cells per well and left under the mentioned conditions. The bacteria were grown for 16 h, 200 rpm, 37°C in LB and sub-cultured (∼10^7^ CFU/mL) into fresh LB and incubated for additional 3 h. At this point, when the bacteria reached an OD600∼0.5, the cultures were centrifuged at 6000 rpm for 5 min. The bacteria were resuspended to OD600 = 1.0 (1×10^9^ bacteria per ml) in 0.9% NaCl and added to monolayers at a multiplicity of infection of 100∶1 (bacteria to eukaryotic cell). Equal inoculum counts were verified by plating on LB agar plates (counts before infection). After 1 h of infection at 37°C, 5% CO_2_, the media was removed and monolayers were washed twice with 0.9% NaCl. At this point, fresh media containing 250 µg/mL amikacin was added to kill extracellular bacteria. The plates were incubated for further 2 h at 37°C, 5% CO_2_. To enumerate invaded bacteria (t = 2 h), cells were washed twice with 0.9% NaCl and lysed in 1 ml 0.1% Triton X-100 (v/v). The viable intracellular bacteria were enumerated by colony counts of lysate dilutions plated on LB agar. The experiments were performed in quadruplicates.

### Statistical analysis

Statistical significance of the differences between the strains was determined using the GraphPad Prism 6.1 program. In all the assays, the statistical significance (P values) was determined by one-way ANOVA analysis with pair-wise comparison of means by using Tukey's multiple comparison test.

## Results

### Genetic structure of the genomic island ST313-GI

**Figure 1:**
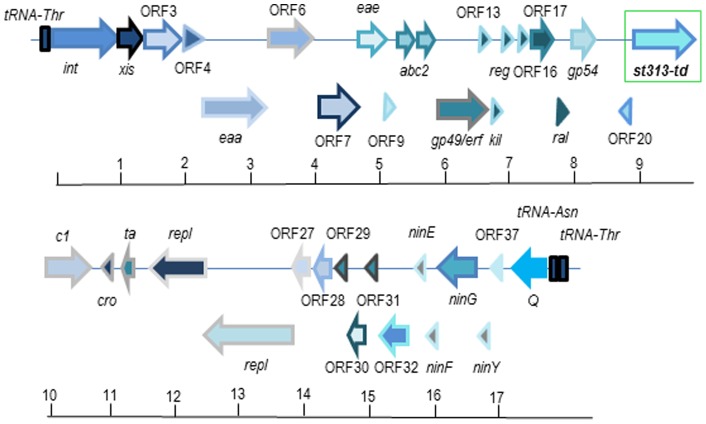
Genetic structure of ST313-GI. The genomic organization is shown with a scale in kb. Arrow above the scale represents predicted ORFs and their transcription directions. ORF 21 corresponds to the virulence gene *st313-td* and is highlighted with a rectangle. Twenty-one ORFs encode phage-related functions: integration and excision: *int* and *xis*, respectively, Ea region: *eaa* and *eae*, recombination: *abc2* (Anti-RecBCD), *erf* and *kil*, regulatory functions: *reg*, structural: *gp54*, anti-restriction: *ral*, inmunity: *c1* (repressor) and *cro* (antirepressor), transcription: *ta* (transcriptional activator), replication: *repl*, Nin region: *nin,* antitermination*: Q* (Q protein). The sequence data have been extracted from the sequenced genome of the strain *S.* Typhimurium D23580, accession no. FN424405.1. The location of ST313-GI within this genome is 368767_386754. The location of *st313-td* is; 377825_378748 (locus_tag = ”STMMW_03531” and protein id: “YP_005231421.1”). All the genes within ST313-GI have been annotated in the genome of D23580. The function of the encoded proteins was determined by using the Uniprot database (http://www.uniprot.org/). See additional Table S2 for more details.

In a previous study we showed that the gene *st313-td* is located within a potential pathogenicity island ST313-GI in *S.* Typhimurium ST313 [12] as illustrated in Figure 1. In the present work, we further characterized this genomic island through *in silico* studies. The sequence of ST313-GI to determine its structure has been extracted from the publicly available and annotated genome of D23580 (accession no. FN424405.1). It is to note that the gene island is not contained within any of the five prophages described in the mentioned genome. ST313-GI, of 17836 bp, is flanked by tRNA genes encoding for threonine (Thr) at the 3′end, and asparagine (Asn) and threonine, at the 5′ end, respectively. Such integration between tRNA encoding genes has typically been described for genomic islands in bacterial chromosomes [47]. In addition, the overall G+C content is 45.7%, which equals that of *Salmonella* phages and other genomic islands [23], [48]. The genomic island ST313-GI consists of 38 Open Reading Frames (ORFs) of which 17 encode unknown functions or predicted phage proteins based on the NCBI annotated analysis and the Uniprot database. Among these 17 ORFs, ORF21 corresponds to the gene *st313-td*. The remaining 21 ORFs of ST313-GI encode phage-related functions, such as replication, integration, excision etc. (Figure 1, Table S2).

### Distribution of the *st313-td* gene among strains of *S.* Typhimurium and *S.* Dublin

The presence of the gene *st313-td* was determined by PCR in 295 *S.* Typhimurium of unknown MLST and 50 *S.* Dublin isolates, which were selected from a global strain collection. Seventy-six (92.7%) out of the 82 *S.* Typhimurium strains from blood specimens exhibited the amplicon corresponding to the gene *st313-td* (Table 1). In contrast, the gene was less frequently found in the 213 *S.* Typhimurium strains collected from other sources and only 45 (21.1%) displayed the band expected for *st313-td*. The latter included 32 and seven isolates originated from stools in Belarus [33] and China [34], respectively and six strains from sewer water in Nepal [35]. It is noteworthy, that *st313-td* gene was absent in the remaining 168 (78.9%) non-blood *S.* Typhimurium isolates, including those collected from animals (n = 73) and food (n = 9) (Table 1). On the other hand, all the 50 (100%) *S.* Dublin strains from human (n = 25) and cattle (n = 25) harboured the *st313-td* gene (Table 1).Thus, results derived from PCR revealed that the gene *st313-td* is mainly associated to invasive strains of *Salmonella*.

### *st313-td* is associated to MLST types of *S.* Typhimurium different from ST313

We assessed whether the non-blood *S.* Typhimurium strains carrying *st313-td* belonged to the lineage ST313. Thus, six non-invasive *S.* Typhimurium isolates representing a wide geographical distribution and positive for the gene *st313-td* were subjected to whole genome sequencing to assign a specific MLST type. None of the six strains from Belarus, China and Nepal belonged to ST313. Thus, the four non-blood isolates from Belarus and Nepal were assigned to the MLST ST328, while the remaining two non-blood isolates from China belonged to ST19 which is one of the predominant *S.* Typhimurium genotype in the MLST database (http://mlst.ucc.ie/). MLST ST313 differs from ST19 at only one MLST locus (*sucA*), which in turns differs from ST328 at the locus *aroC* [49]. These data indicate certain genetically relatedness among the isolates. A SNP tree showing the relatedness/divergence between the representative strains assigned to the different MLST types of *S.* Typhimurium carrying *st313-td* is illustrated in Figure S2. The analysis revealed that the strains clustered in two separate groups. One group included strains of African origin (assigned to ST313) and the second group contained strains from Asia and Europe (assigned to ST19 or ST328). These two groups of strains could either have a common *st313-td* ancestor or the gene could have been transferred independently into the two groups from another source.

### Analysis of the expression of the gene *st313-td*

In order to study whether the *st313-td* gene was actually expressed in the different isolates where we had detected the gene, RT-qPCR was performed on a selection of strains. The collection included; two strains of *S.* Dublin (from cattle and human, respectively) and 12 strains of *S.* Typhimurium (two from each of the countries where *st313-td*-positive isolates were identified and belonging to the ST types so far detected as carriers of *st313-td*; ST313, ST19 and ST328) (Table 1, Figure S2). The gene was shown to be expressed in all the strains tested at early logarithmic phase of growth in LB (data not shown).

### The genomic island ST313-GI containing st313-td is apparently restricted to *S.* Typhimurium ST313

To study whether *st313-td* was always harboured within ST313-GI, *in silico* studies were performed to determine the presence of the gene island in the publicly available genomes containing the gene. In a previous study, ST313-GI was shown to be restricted to S. Typhimurium ST313 strains collected in sub-Saharan Africa [12] (unpublished data). Thus, ST313-GI was not found in the two *S.* Dublin chromosomes available in Genbank (accession no. CP001143 and CM001151). In these genomes, only a fragment of ∼6.8 kb of ST313-GI carrying *st313-td* was detected [12].

In the six genomes of isolates from Belarus (n = 2), China (n = 2) and Nepal (n = 2) harbouring the *st313-td* gene and analysed by whole genome sequencing in the present work, only a fraction of ST313-GI was present. The ST313-GI fragments differed in size between the strains tested: a fragment of 1467 bp was detected in strains from Belarus, of 4245 bp in strains from China, and of 11317 bp in strains from Nepal. Therefore, the presence of the entire ST313-GI seems to be restricted to the *S.* Typhimurium ST313 lineage circulating in sub-Saharan Africa.

### Deletion of *st313-td* causes virulence attenuation in systemic mice infection

In order to investigate whether *st313-td* plays a role in virulence, the mutant strain, Δ*st313-td* was tested in competition with the wild type strain in a mouse model of systemic infection. Interestingly, the deletion of *st313-td* decreased virulence in C57/BL6 mice (CI: 0.0124; P value <0.001) (Table 3). Introduction of the plasmid pAHF4 (encoding a cloned copy of the gene) to the mutant *in trans,* restored, partly, the virulence to the wild type level and removed significant differences between the two strains (CI: 0.3340; P value >0.05) (Table 3). Besides, no significant difference was found between the complemented and the WT strains (P value >0.05). These data indicate that the increase in virulence was indeed caused by the *st313-td* mutation. In contrast, the CI of the mutant 4/74-C (harbouring *st313-td*) versus the WT 4/74 was slightly increased, yet not significantly (CI: 1.5251; P value >0.05) (Table 3).

**Table 3:** Competitive index analysis of *S*. Typhimurium mutants.

*Salmonella* strains	Competitive index (CI)
WT 02/03–002	
versus	
Δ*st313-td* (4)	0,0124^a^
02-03/002-C (4)	0,3340^b^
WT 4/74	
versus	
4/74-C (4)	1,5251^a^

CI = 1: the virulence of the strains tested is equal.

CI<1: the mutant is less virulent than the WT.

^a^ CI was significantly different from 1.0 (P value <0.001).

^b^ CI was significantly different from corresponding mutant (P value >0.05).

Competition indices were calculated based on input (CFU/mL of inoculum) and output.

(CFU/mL of spleen sample) numbers of wild type versus mutant bacteria as previously.

described [43]. The results are shown as means values based on the number of mice tested.

(indicated in brackets).

Virulence testing was based on competition for success between WT and mutant in the same animal, as suggested by Beuzon *et al.* [50] as a way to increase sensitivity in virulence testing since mouse-to-mouse variation is minimized.

### *st313-td* contributes to *S.* Typhimurium intracellular survival within murine macrophages

**Figure 2:**
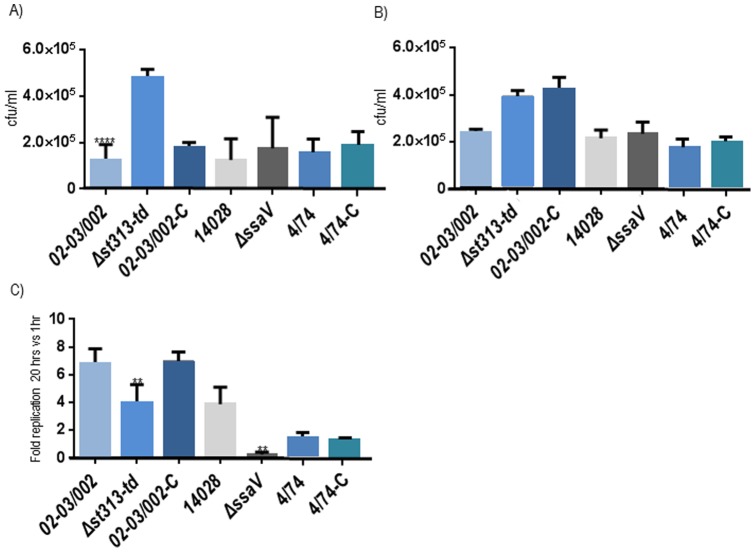
Infection of J774 mouse macrophages. Intracellular survival/replication, A) at t = 1 h post-infection with opsonisation, B) at t = 1 h post-infection without opsonisation, and C) at t = 20 h post-infection with opsonisation, and with regards to t = 1 h post-uptake (the intracellular survival at t = 1 h for all the tested isolates is regarded as 1.0). Strains tested were: *S.* Typhimurium WT 02–03/002, 4/74 and 14028 and the mutant isolates: Δ*st313-td*, 02-03/002-C (pAHF4, complemented strain), 4/74-C (pAHF4) and Δ*ssaV*. The experiments were repeated at least 4 times with similar results and shown is an average of these. Errorbars indicate standard deviation. Statistical significance (****  = P<0,00001; **  = P<0,001) was determined by one-way ANOVA analysis with pair-wise comparison of means.

Virulence of *S*. Typhimurium in the mouse model of systemic disease was reduced in the absence of *st313-td*. In order to study what might have caused this decrease, the ability of the mutant and complemented strains to infect and survive inside J774 macrophages was analysed. In the macrophage experiments where the strains were complement-opsonized, the WT 02–03/002 showed significantly (P value <0.05) less intracellular counts at t = 1 h post-infection than the mutant isolate Δ*st313-td* (Figure 2A). On the other hand, no statistical significant difference was observed between the WT 4/74 and the construct, 4/74-C, nor between the three parental strains 02–03/002, 4/74 and 14028.

After t = 20 h post-uptake, Δ*st313-td* was significantly (3.5 fold) reduced (P value <0.05) compared to the WT 02–03/002. Interestingly, at this time point, the WT 02–03/002 and the complemented, 02-03/002-C, strains showed a similar net replication of ∼7 significantly higher than observed for the isolates 4/74 and 4/74-C (Figure 2C). Notably, no significant difference in intracellular survival/replication was observed between the WT 4/74 and the mutant, 4/74-C, isolates at any time point post-infection (Figure 2A and 2C).

In order to investigate whether opsonisation could affect the uptake depending on the presence/absence of the gene *st313-td*, similar experiments were conducted with no bacterial opsonisation. In this experiments, no significant difference (P value >0.05) was observed between the WT 02/03–002 and the mutant Δ*st313-td* at 1 hour post challenge (Figure 2B) indicating that the gene improves uptake by activated macrophages and/or survival inside these cells. In these experiments intracellular survival was also checked at time t = 20 h and the trend was the same than in the assays where opsonisation was performed (not shown).

The mutant in the SPI-2 gene *ssaV* which is attenuated for macrophage survival [45] was included as control, and showed the expected phenotype, as it was taken up to the same extend as the WT strain, but showed reduced intracellular propagation (Figure 2C).

It was also confirmed (P value >0.05) that the phenotype of the WT 02/03–002 and 4/74 was not affected by the introduction of the plasmid pACY177 used for complementation (data not shown).

**Figure 3:**
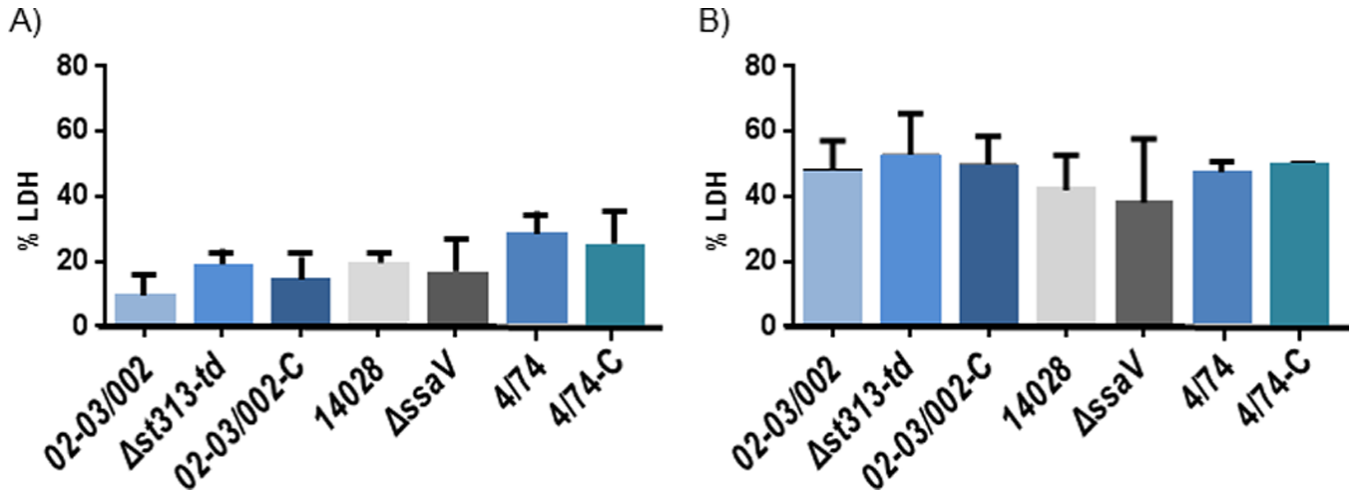
Cytotoxicity towards J774 mouse macrophages. Measurement of cytotoxicity levels, A) at t = 1 h post-infection and B) t = 20 h post-infection without opsonization, respectively. Strains tested for cytotoxicity were: *S.* Typhimurium WT 02–03/002, 4/74 and 14028 and the mutant isolates: Δ*st313-td*, 02-03/002-C (pAHF4, complemented strain), 4/74-C (pAHF4) and Δ*ssaV.* The citotoxicity level is based on the release of LDH (lactate dehydrogenase). The experiments were repeated at least four times with similar results and shown is an average of these. Errorbars indicate standard deviation. Statistical significance was determined by one-way ANOVA analysis with pair-wise comparison of means.

Theoretically, since cytotoxic effects results in exposure to the antibiotic in such cell culture experiments, a strain with increased multiplication ability could be masked by an increase in cytotoxicity. However, the mutated strains did not differ significantly from the WT in cytotoxicity 20 h post infection (P value >0.05) (Figure 3B). Same results were observed at t = 1 post- uptake where all the strains tested showed similar cytotoxicity levels (Figure 3A).

### *st313-td* is not needed for invasion of the epithelial cells

**Figure 4:**
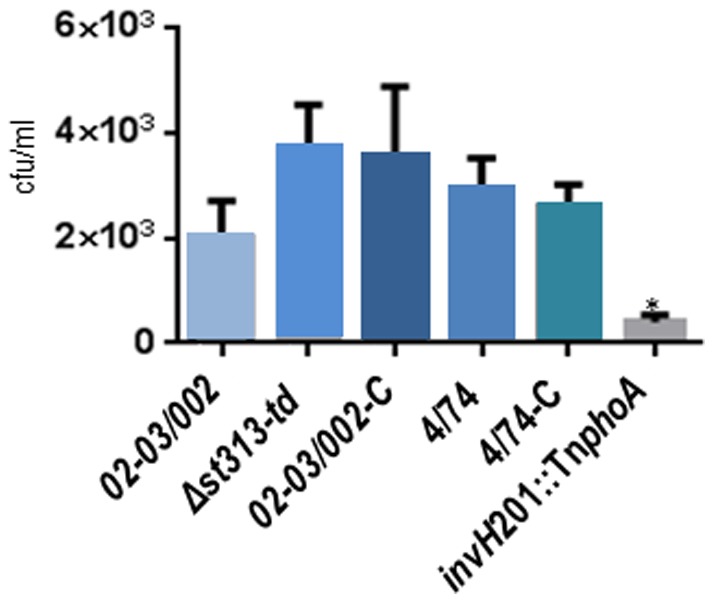
Infection of the human epithelial Int407 cells. Invasion of monolayes of Int407 cells was performed and invaded bacteria were enumerated at t = 2 h post-infection. Strains tested were: *S.* Typhimurium WT 02–03/002 and 4/74, and the mutant isolates: Δ*st313-td*, 02-03/002-C (pAHF4, complemented strain), 4/74-C (pAHF4) and *invH*201::Tn*phoA.* The experiments were repeated at least 4 times with similar results and shown is an average of these. Error bars indicate standard deviation. Statistical significance was determined by one-way ANOVA analysis with pair-wise comparison of means.

Infection of the intestinal epithelial layer is the first critical step in *Salmonella* virulence. This aspect of infection was bypassed in our mice experiments, since we used intra peritoneal challenge. We therefore tested the role of *st313-td* in invasion in an epithelial cell infection model. The gene *st313-td* was not involved in invasion of the human epithelial INT-407 cells (Figure 4) since its deletion did not change the ability of *S*. Typhimurium to invade these cell lines compared to the WT, whereas the control strain *invH*201::Tn*phoA*, a *S*. Typhimurium strain with a deficiency in T3SS1 [46], was decreased in this phenotype (P value <0.01). The presence of the plasmid pACY177 in the WT strain 02–03/002 did not significantly (P value >0.05) affect its invasion rate (not shown).

## Discussion

A distinct genotype of *S.* Typhimurium, ST313, has emerged in sub-Saharan Africa and might have adapted to cause potentially fatal bacteraemia in humans. To better understand the underlying virulence mechanisms and to identify the possible factors responsible for its increased pathogenicity compared to other clones of *S.* Typhimurium is of paramount importance for human health.

A gene, *st313-td,* has recently been detected in the chromosomes of the *S.* Typhimurium ST313 and *S.* Dublin isolates through comparative genomics [12]. Pimlapas *et al*. [12] reported a total of 18 *S.* Typhimurium strains from DRC and Nigeria assigned to ST313 carrying *st313-td*. The gene was harboured in a novel putative pathogenicity island of ∼18 kb in *S.* Typhimurium ST313, termed ST313-GI [12].

In this study, we assessed the role of the gene of yet unknown function, *st313-td,* in *S.* Typhimurium ST313 virulence by using *in vitro* and *in vivo* experiments.

To study the role of *st313-td* in *S.* Typhimurium virulence, mice experiments were performed. *S.* Typhimurium causes invasive disease in the mouse and thus, this animal model is useful for studying the role of the gene in systemic *Salmonella* infection.

Interestingly, an evident role of *st313-td* in systemic infection was found and the WT 02–03/002 clearly outcompeted the mutant Δ*st313-td*. No significant increase in virulence was observed for the mutant strain, 4/74-C (harbouring *st313-td*) compared to the WT 4/74 however, its CI increased by 50%. The data derived from mice experiments showed that the mutant, Δ*st313-td,* is significantly less pathogenic than the WT, suggesting that *st313-td* contributes to virulence during the systemic infection in mice. The results from the mice experiments performed with the WT 4/74 and the mutant 4/74-C must be regarded as inconclusive, but probably other factors (apart from this encoded by *st313-td*) not present in WT 4/74 may be required to lead to an increase in virulence.

Additionally, infection of mice macrophages and human epithelial cell lines were performed to further detail the virulence enhancing role of *st313-td*. These assays were considered relevant because infection and intracellular survival in macrophages is required for full virulence and infection of the intestinal epithelial layer is the first critical step in *Salmonella* virulence [51], [52]. Surprisingly, the lack of *st313-td* caused an increase on numbers of intracellular bacteria in macrophages at t = 1 h post-infection, but only in experiments where opsonisation was performed. This indicates that *st313-td* influences the uptake and/or early survival in macrophages but only in activated cells. Notably, in a previous study it was demonstrated that *S.* Typhimurium D23580, assigned to ST313 and carrying *st313-td* requires high levels of complement for antibody-mediated bactericidal activity compared with *S.* Typhimurium LT2 [19]. Thus, *st313-td* might play a role in virulence by either decreasing the binding of the bacteria to antibodies causing less uptake in macrophages or by diminishing the susceptibility to complement mediated lysis. Further studies are required to confirm this hypothesis.

Once inside the macrophages, lack of *st313-td* caused a significantly decreased intracellular survival. It is well known that *Salmonella* are facultative intracellular bacteria and their ability to survive within cells is essential for virulence [53]. Therefore, the gene *st313-td* appears to encode a factor that favours the persistence of intracellular *Salmonella*. This trait may explain the virulence enhancing effect in the mice studies. These data indicate that the virulence enhancing role of *st313-td* appears to be dual and related both to a decreased uptake of bacteria in phagocytic cells and a better survival at late time points. Both these phenotypes may add to a higher ability to cause systemic infection, which is a hall mark of ST313. Results of cell culture infection can be influenced by levels of toxicity, since bacteria that kill the macrophages subject themselves to killing by antibiotics in the media. It is therefore important to note that strains tested in the macrophage infections showed similar cytotoxicity levels at t = 20 h.

Contrary to the results using macrophages, deletion of *st313-td* did not influence invasion of epithelial cells. Thus the function of the gene product seems to be specific for phagocytic cells, and hence only relevant for systemic infection.

Attempts to delete the whole genomic island ST313-GI were performed several times as part of the current study with no success. These data suggest that the carriage of ST313-GI in *S.* Typhimurium ST313 is essential during the conditions used for mutagenesis.

A strong association of the gene *st313-td* to isolates of *S.* Typhimurium from cases of bacteraemia was observed by PCR and furthermore, *S.* Dublin, which is a serovar highly invasive in humans [54] was always found to carry *st313-td*. The gene was also shown to be expressed in a collection of both *S.* Dublin and *S.* Typhimurium strains. These data support a potential role of *st313-td* in systemic salmonellosis caused by these serovars.

Taking into account that all the *S.* Dublin isolates proved to harbour the gene, this serovar is a likely ancestor of the gene *st313-td*, subsequently transferred to *S.* Typhimurium. Further analysis should be performed in order to confirm this speculation and to investigate whether it plays a role in *S.* Dublin virulence.

The presence of *st313-td* in a genetic mobile element, the genomic island ST313-GI, supports our hypothesis that the gene might have been transferred by horizontal gene transfer to *S.* Typhimurium. It is noteworthy that Wain *et al*. [13] suggested that *S*. Typhimurium ST313 likely emerged from a pathogen which was already adapted to an animal host, causing diarrhoea in healthy humans and invasive disease in HIV-infected patients as a result of their immune deficiency.

The horizontal transfer of *st313-td* is also supported by the fact that six strains isolated from human stools in Belarus and China, and from water in Nepal assigned to other ST types (ST328, ST19 and ST328, respectively) than ST313 also carried the gene. However, in contrast to the ST313 strains, the six non-ST313 isolates analysed did not harbour the entire ST313-GI and only small portions of the genomic island matched the genomes of these strains. Therefore, contrary to the situation observed for the single gene *st313-td*, the genomic island ST313-GI appears to be currently restricted to the clade ST313.

While ST313 has been specifically localized to the sub-Saharan Africa, ST19 is the most commonly identified *S.* Typhimurium sequence type and is globally predominating [10]. Both sequences types are circulating simultaneously in Kenya [10] and co-circulation might allow genetic exchange and could explain the spread of *st313-td* among the two clonal groups. These results reveal that strains harbouring the gene are not restricted to the clonal group ST313 and sub-Saharan Africa. Recently, it has been suggested that birds might have been a vehicle or source of *S*. Typhimurium ST313 [12], and if this is true, a wider spread of the gene is to be expected.

Interestingly, *st313-td* was not detected in any *S.* Typhimurium strain of food or animal origin. Thus, in *S.* Typhimurium, the apparently restricted presence of *st313-td* in human isolates suggests a potential host specificity for this gene as suggested for its carrier, *S.* Typhimurium ST313. In a previous work, the authors showed reduced genome in the *S*. Typhimurium ST313 strain D23580, in comparison to gastroenteritis strains, a finding consistent with what might be expected of strains undergoing host adaptation [12].

We demonstrated that a *st313-td* mutant displayed diminished survival within J774 macrophages and was attenuated in virulence in a systemic murine infection. Thus, this work adds another chapter to understanding the virulence in *S.* Typhimurium but also highlights the need for a closer monitoring of this sequence type to control it and to avoid a further spread. Further work should be carried out for a deepest characterization of the genomic island and its possible essentiality in strains of ST313. Apart from immunity studies, prevention of NTS bacteraemia also requires genetic studies analysing the diversity of the circulating invasive strains. These studies contribute to investigate the epidemiology and transmission of this African pathogen, allow a better understanding of the pathogenesis of the NTS disease and represent key approaches for the developing of a much needed vaccine as there are limited antibiotic choices against NTS infections.

## Supporting Information

Figure S1 **SNP tree showing the divergence/relatedness among the strains representing the identified ST types carrying** ***st313-td.*** The reference strain *S.* Typhimurium D23580 and the ST313 isolate used in the present study 02–03/002 are highlighted in bold. Countries of origin and ST types are also indicated.
(TIF)

Table S1 Primers designed and used in this work.
(DOCX)

Table S2 Characteristics of the proteins encoded by the ST313-GI. Sequence data (ORFs) extracted from the genome of the *Salmonella* Typhimurium D23580 available at NCBI (accession no. FN424405.1). Uniprot database was used to determine the function of the encoded proteins.
(DOCX)
