# Supplementary figures and images for: The Role of the st313-td Gene in Virulence of Salmonella Typhimurium ST313
Source: PLoS One. 2014 Jan 3;9(1):e84566. doi: 10.1371/journal.pone.0084566 (PMC3880295; doi:10.1371/journal.pone.0084566)

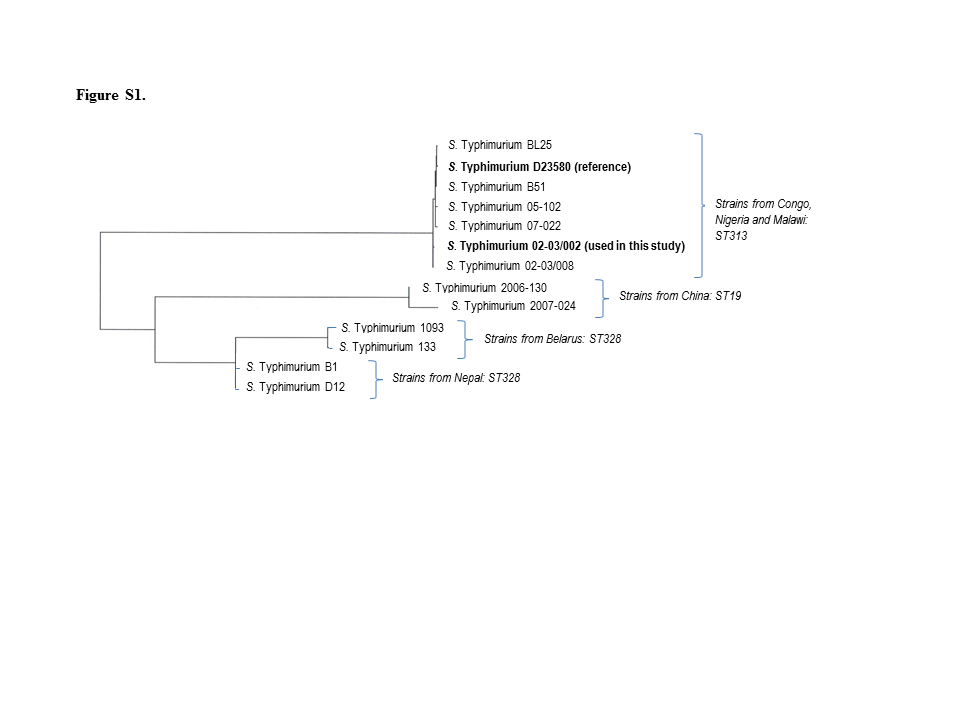

Supplement: Figure S1 — SNP tree showing the divergence/relatedness among the strains representing the identified ST types carrying st313-td. The reference strain S. Typhimurium D23580 and the ST313 isolate used in the present study 02–03/002 are highlighted in bold. Countries of origin and ST types are also indicated. (TIF) [file pone.0084566.s001.tif]
