# Supplementary material for: The Role of the st313-td Gene in Virulence of Salmonella Typhimurium ST313
Source: PLoS One. 2014 Jan 3;9(1):e84566. doi: 10.1371/journal.pone.0084566 (PMC3880295; doi:10.1371/journal.pone.0084566)
Supplement: Table S1 — Primers designed and used in this work. (DOCX) [file pone.0084566.s002.docx]

**Table S1**

| **Primer no.** | **Sequence^a^** | **Amplicon size (bp)** |
| --- | --- | --- |
| *Primers for the screening of st313-td in the Salmonella strains* | | |
| ST313-td-F  ST313-td-R | 5´-GGTGATGTAGAAAGACGAA-3´  5´-GGAAGAGTAGGGAAAAGAA-3´ | 1029 |
| *Primers for cloning* | | |
| ST313-Sca1-F  ST313-Pst1-R | 5`-CCCAGTACTGGTGATGTAGAAAGACGAA -3´  5`- CCCCTGCAGGGAAGAGTAGGGAAAAGAA -3´ | 1047 |
| *Primers for mutagenesis* | | |
| ST313-F_KO  ST313-R_KO | 5`-CGTTAGCGCTTGCCCGCCACACTTTAACAAGGAAAATCAA*TCTGCAGCTCACGGTAACTG-3´*  5`-AGTAGGGAAAAGAAAACCCGGCGCTGTGGCCGGGCGTTGA*CGCTGTCGAACTTTTCGATC* -3´ | 1328 |
| *Primers for expression studies* | | |
| ST313-F-RTqPCR  ST313-R-RTqPCR | 5`-GCCGCTTACTGGATTTCTTG*-3´*  5`-AGCGGGACCATTATCCATAC*-3´* | 133 |

^a^The restriction endonuclease sites engineered into the primer sequences are underlined. AGTACT: *Sca*I, CTGCAG: *Pst*I. Letters in italics indicate primer sequences that annealed with *aac(3)IV*-resistance cassette from pUO9090 [M.C. Martin, unpublished results]. The expected amplicon for this cassette is 1288 bp
