# Supplementary material for: The Role of the st313-td Gene in Virulence of Salmonella Typhimurium ST313
Source: PLoS One. 2014 Jan 3;9(1):e84566. doi: 10.1371/journal.pone.0084566 (PMC3880295; doi:10.1371/journal.pone.0084566)
Supplement: Table S2 — Characteristics of the proteins encoded by the ST313-GI. Sequence data (ORFs) extracted from the genome of the Salmonella Typhimurium D23580 available at NCBI (accession no. FN424405.1). Uniprot database was used to determine the function of the encoded proteins. (DOCX) [file pone.0084566.s003.docx]

**Table S2**

| **Uniprot Entry (ORF)** | **protein name/product** | **Organism** | **gene name** |
| --- | --- | --- | --- |
| >CDS_369852-368857 (1) |  |  |  |
| C9X830_SALTD | Integrase | Salmonella typhimurium (strain D23580) | STMMW_03341 |
|  |  |  |  |
| >CDS_370247-369897 (2) |  |  |  |
| C9X831_SALTD | Excisionase | Salmonella typhimurium (strain D23580) | STMMW_03351 |
| VXIS_BPP22 | Excisionase | Enterobacteria phage P22 (Bacteriophage P22) | xis |
|  |  |  |  |
| >CDS_370851-370258 (3) |  |  |  |
| C9X832_SALTD | Predicted prophage protein | Salmonella typhimurium (strain D23580) | STMMW_03361 |
|  |  |  |  |
| >CDS_371207-370923 (4) |  |  |  |
| C9X833_SALTD | Similar to bacteriophage 933W L0064 | Salmonella typhimurium (strain D23580) | STMMW_03371 |
|  |  |  |  |
| >CDS_372165-371200 (5) |  |  |  |
| C9X834_SALTD | Predicted prophage protein | Salmonella typhimurium (strain D23580) | STMMW_03381 |
| VEAA_BPP22 | Eaa protein | Enterobacteria phage P22 (Bacteriophage P22) | eaa |
|  |  |  |  |
| >CDS_372931-372176 (6) |  |  |  |
| C9X835_SALTD | Predicted prophage protein | Salmonella typhimurium (strain D23580) | STMMW_03391 |
|  |  |  |  |
| >CDS_373575-372931 (7) |  |  |  |
| C9X836_SALTD | Predicted prophage protein | Salmonella typhimurium (strain D23580) | STMMW_03411 |
|  |  |  |  |
| >CDS_373982-373572 (8) |  |  |  |
| C9X837_SALTD | Predicted prophage protein | Salmonella typhimurium (strain D23580) | STMMW_03421 |
| G9TY48_SALMO | Eae protein | Salmonella enterica subsp. enterica serovar Montevideo str. LQC 10 | SEEM010_00732 |
|  |  |  |  |
| >CDS_374149-373979 (9) |  |  |  |
| C9X838_SALTD | Predicted prophage protein | Salmonella typhimurium (strain D23580) | STMMW_03422 |
| L7AK16_SALET | Prophage protein | Salmonella enterica subsp. enterica serovar Agona str. SH08SF124 | F514_22317 |
|  |  |  |  |
| >CDS_374453-374160 (10) |  |  |  |
| C9X839_SALTD | Anti-RecBCD | Salmonella typhimurium (strain D23580) | STMMW_03431 |
| ABC2_BPP22 | Anti-RecBCD protein 2 | Enterobacteria phage P22 (Bacteriophage P22) | abc2 |
|  |  |  |  |
| >CDS_374784-374500 (11) |  |  |  |
| C9X840_SALTD | Anti-RecBCD | Salmonella typhimurium (strain D23580) | STMMW_03441 |
| Q5PF59_SALPA | Putative uncharacterized protein abc1 | Salmonella paratyphi A (strain ATCC 9150 / SARB42) | abc1 SPA2424 |
|  |  |  |  |
| >CDS_375491-374784 (12) |  |  |  |
| G9VFU0_SALMO | Gp49 protein | Salmonella enterica subsp. enterica serovar Montevideo str. 4441 H | SEEM41H_03232 |
| I6R0N0_9CAUD | Erf | Salmonella phage vB_SemP_Emek | erf |
|  |  |  |  |
| >CDS_375631-375488 (13) |  |  |  |
| C9X842_SALTD | Predicted prophage protein | Salmonella typhimurium (strain D23580) | STMMW_03461 |
|  |  |  |  |
| >CDS_375809-375621 (14) |  |  |  |
| C9X843_SALTD | Kil | Salmonella typhimurium (strain D23580) | STMMW_03471 |
| B8K1E1_9CAUD | Kil | Salmonella phage SE1 | orf17 |
|  |  |  |  |
| >CDS_375972-375790 (15) |  |  |  |
| C9X844_SALTD | Regulatory protein | Salmonella typhimurium (strain D23580) | STMMW_03481 |
| I6R0R7_9CAUD | Putative CIII protein | Salmonella phage vB_SosS_Oslo |  |
|  |  |  |  |
| >CDS_376205-376059 (16) |  |  |  |
| C9X845_SALTD | Predicted prophage protein | Salmonella typhimurium (strain D23580) | STMMW_03491 |
| SECY2_LACJO | Accessory Sec system protein translocase subunit | Lactobacillus johnsonii (strain CNCM I-12250 / La1 / NCC 533) | secY2 LJ_0384 |
|  |  |  |  |
| >CDS_376565-376266 (17) |  |  |  |
| C9X846_SALTD | Predicted prophage protein | Salmonella typhimurium (strain D23580) | STMMW_03501 |
|  |  |  |  |
| >CDS_376805-376605 (18) |  |  |  |
| C9X847_SALTD | Antirestriction protein | Salmonella typhimurium (strain D23580) | STMMW_03511 |
| VRAL_BPP22 | Restriction inhibitor protein ral | Enterobacteria phage P22 (Bacteriophage P22) | ral |
|  |  |  |  |
| >CDS_377216-376884 (19) |  |  |  |
| B5FLS5_SALDC | Gp54 | Salmonella dublin (strain CT_02021853) | STMMW_03511 |
| H1RCV7_SALMO | Gp54 | Salmonella enterica subsp. enterica serovar Montevideo str. IA_2010008286 | SEEM8286_01974 |
|  |  |  |  |
| >CDS_377619-377789 (20) |  |  |  |
| C9X849_SALTD | Predicted prophage protein | Salmonella typhimurium (strain D23580) | STMMW_03522 |
|  |  |  |  |
| >CDS_378748-377825 (21) |  |  |  |
| C9X850_SALTD | Predicted prophage protein | Salmonella typhimurium (strain D23580) | STMMW_03531 |
|  |  |  |  |
| >CDS_379562-378837 (22) |  |  |  |
| C9X851_SALTD | Repressor protein cI | Salmonella typhimurium (strain D23580) | STMMW_03541 |
| Q5PF51_SALPA | Transcriptional activator-regulatory protein | Salmonella paratyphi A (strain ATCC 9150 / SARB42) | gp C1 SPA2414 |
|  |  |  |  |
| >CDS_379637-379852 (23) |  |  |  |
| C9X852_SALTD | Antirepressor protein | Salmonella typhimurium (strain D23580) | STMMW_03551 |
| K0Q7Y9_SALNE | Phage transcriptional regulatory protein | Salmonella enterica subsp. enterica serovar Newport str. Levine 1 |  |
| Q76H55_9CAUD | Cro | Enterobacteria phage ST104 | cro |
|  |  |  |  |
| >CDS_379963-380244 (24) |  |  |  |
| C9X853_SALTD | Transcriptioanl activator | Salmonella typhimurium (strain D23580) | STMMW_03561 |
| F2FI89_SALDU | C1 phage protein | Salmonella enterica subsp. enterica serovar Dublin str. SD3246 | SD3246_0610 |
|  |  |  |  |
| >CDS_380418-381251 (25) |  |  |  |
| C9X854_SALTD | Replication protein | Salmonella typhimurium (strain D23580) | STMMW_03571 |
| B5FLT2_SALDC | DNA replication protein gp18 | Salmonella dublin (strain CT_02021853) |  |
|  |  |  |  |
| >CDS_381248-382624 (26) |  |  |  |
| C9X855_SALTD | Replication protein | Salmonella typhimurium (strain D23580) | STMMW_03581 |
| Q76H51_9CAUD | 12 | Enterobacteria phage ST104 | 12 |
|  |  |  |  |
| >CDS_382621-382890 (27) |  |  |  |
| C9X856_SALTD | Predicted prophage protein | Salmonella typhimurium (strain D23580) | STMMW_03582 |
| G5QFQ0_SALRU | Phage protein | Salmonella enterica subsp. enterica serovar Rubislaw str. A4-653 | LTSERUB_1198 |
|  |  |  |  |
| >CDS_382962-383234 (28) |  |  |  |
| C9X857_SALTD | Predicted prophage protein | Salmonella typhimurium (strain D23580) | STMMW_03601 |
| Q8HAG2_BPST6 | Orf-90 | Enterobacteria phage ST64T (Bacteriophage ST64T) | orf-90 |
|  |  |  |  |
| >CDS_383244-383453 (29) |  |  |  |
| C9X858_SALTD | Predicted prophage protein | Salmonella typhimurium (strain D23580) | STMMW_03602 |
| Q8HAG1_BPST6 | Orf-69 | Enterobacteria phage ST64T (Bacteriophage ST64T) | orf-69 |
|  |  |  |  |
| >CDS_383450-383758 (30) |  |  |  |
| C9X859_SALTD | Predicted prophage protein | Salmonella typhimurium (strain D23580) | STMMW_03611 |
| Q8HAG0_BPST6 | Orf-86 | Enterobacteria phage ST64T (Bacteriophage ST64T) | orf-86 |
|  |  |  |  |
| >CDS_383755-383949 (31) |  |  |  |
| C9X860_SALTD | Predicted prophage protein | Salmonella typhimurium (strain D23580) | STMMW_03621 |
| L7ALQ1_SALET | Prophage protein | Salmonella enterica subsp. enterica serovar Agona str. SH08SF124 | F514_22437 |
|  |  |  |  |
| >CDS_383906-384352 (32) |  |  |  |
| C9X861_SALTD | Predicted prophage protein | Salmonella typhimurium (strain D23580) | STMMW_03631 |
|  |  |  |  |
| >CDS_384489-384671 (33) |  |  |  |
| C9X862_SALTD | Predicted prophage protein | Salmonella typhimurium (strain D23580) | STMMW_03641 |
| C6ZR57_9CAUD | NinE | Salmonella phage g341c | ninE |
|  |  |  |  |
| >CDS_384668-384838 (34) |  |  |  |
| C9X863_SALTD | Predicted prophage protein | Salmonella typhimurium (strain D23580) | STMMW_03651 |
| NINF_BPP21 | Protein ninF | Enterobacteria phage P21 (Bacteriophage 21) (Bacteriophage P21) | ninF |
|  |  |  |  |
| >CDS_384831-385442 (35) |  |  |  |
| C9X864_SALTD | Predicted prophage protein | Salmonella typhimurium (strain D23580) | STMMW_03661 |
| A3EYW9_9CAUD | NinG | Salmonella phage SETP10 | ninG |
|  |  |  |  |
| >CDS_385439-385663 (36) |  |  |  |
| C9X865_SALTD | Predicted prophage protein | Salmonella typhimurium (strain D23580) | STMMW_03671 |
| NINY_BPP22 | Putative protein ninY | Enterobacteria phage P22 (Bacteriophage P22) | ninY |
| K5ASV7_SALET | Phage NinY | Salmonella enterica subsp. enterica serovar Heidelberg str. CFSAN00328 | CFSAN00328_10471 |
|  |  |  |  |
| >CDS_385660-385863 (37) |  |  |  |
| C9X866_SALTD | Predicted prophage protein | Salmonella typhimurium (strain D23580) | STMMW_03681 |
|  |  |  |  |
| >CDS_386020-386538 (38) |  |  |  |
| C9X867_SALTD | Predicted antiterminator protein | Salmonella typhimurium (strain D23580) | STMMW_03691 |
| L3L9Z2_ECOLX | Phage antitermination protein | Escherichia coli KTE53 | A1SE_01153 |
| D2A7X4_SHIF2 | Q protein | Shigella flexneri serotype X (strain 2002017) | SFxv_0332 |
| Q8HAE9_BPST6 | Gp23 | Enterobacteria phage ST64T (Bacteriophage ST64T) |  |
